# N‐terminal pro‐B‐type natriuretic peptide improves the predictive value of CHA_2_DS_2_‐VASc risk score for long‐term cardiovascular events in acute coronary syndrome patients with atrial fibrillation

**DOI:** 10.1002/clc.24037

**Published:** 2023-05-22

**Authors:** Xuefei Mu, Miaohan Qiu, Yi Li, Ziqi Li, Bin Qi, Zilan Jing, Quanmin Jing

**Affiliations:** ^1^ Department of Cardiology, General Hospital of Northern Theater Command Cardiovascular Research Institute Shenyang China

**Keywords:** acute coronary syndrome, atrial fibrillation, CHA_2_DS_2_‐VASc score, NT‐proBNP, prognosis

## Abstract

**Background:**

It is important to identify patients with co‐morbid acute coronary syndrome (ACS) and atrial fibrillation (AF) at high risk and adopt proper management strategies to improve their prognosis.

**Hypothesis:**

The addition of N‐terminal pro‐B‐type natriuretic peptide (NT‐proBNP) could improve predictive value for long‐term cardiovascular events beyond the CHA_2_DS_2_‐VASc score in patients with co‐morbid ACS and AF.

**Methods:**

A total of 1223 patients with baseline NT‐proBNP between January 2016 and December 2019 were included in the study. The primary endpoint was all‐cause death at 12 months. The secondary outcomes included 12‐month cardiac death and major adverse cardiovascular and cerebrovascular event (MACCE), defined as a composite of all‐cause death, myocardial infarction, or stroke.

**Results:**

A higher serum of NT‐proBNP levels was strongly associated with increased risks of all‐cause death (adjusted hazard ratio [HR]: 1.05, 95% confidence interval [CI], 1.03–1.07), cardiac death (adjusted HR: 1.05, 95% CI, 1.03–1.07), and MACCE (adjusted HR: 1.04, 95% CI, 1.02–1.06). The prognostic accuracy of the CHA_2_DS_2_‐VASc score was improved when combined with NT‐proBNP to yield a 9%, 11%, and 7% increment for the discrimination of long‐term risk for all‐cause mortality (area under curve [AUC]: from 0.64 to 0.73), cardiac death (AUC: from 0.65 to 0.76), and MACCE (AUC: from 0.62 to 0.69), respectively.

**Conclusions:**

In patients with ACS and AF, NT‐proBNP is a potential biomarker to enhance risk discrimination for all‐cause death, cardiac death, and MACCE in combination with the CHA_2_DS_2_‐VASc score.

## Graphical abstract

N‐terminal pro‐B‐type natriuretic peptide (NT‐proBNP) is a strong risk factor for poor prognosis and enables a more accurate appreciation of risk on top of the CHA_2_DS_2_‐VASc score in patients co‐morbid acute coronary syndrome and atrial fibrillation.

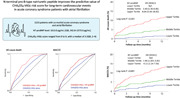

## 1 INTRODUCTION

Atrial fibrillation (AF) remains a commonly encountered complication in acute coronary syndrome (ACS) patients.^1^ With a significant overlap of risk factors, concomitant AF may result in short‐ and long‐term worse clinical outcomes in patients with ACS.^2^, ^3^ It is, therefore, important to identify individuals at high risk and adopt proper management strategies to improve their prognosis. In this regard, current guidelines recommend the use of CHA_2_DS_2_‐VASc risk score for risk assessment and subsequent treatment guidance in patients with co‐morbid ACS and AF.^4^, ^5^ However, the score is solely based on clinical parameters, including the presence of congestive heart failure (HF), hypertension, age, diabetes mellitus, history of stroke, vascular disease, and sex.^6^ Moreover, a physician‐based survey identified knowledge gaps in the application of the score for lack of a clear definition for its components.^7^ For this reason, the algorithm by adding laboratory‐assessed biomarkers to the CHA_2_DS_2_‐VASc score might be more objective and precision to predict the risk of adverse events.

N‐terminal pro‐B‐type natriuretic peptide (NT‐proBNP) is a nonactive prohormone that is released from the same molecule that produces BNP, mainly in response to changes in cardiac structure and function. Previous studies demonstrated a higher serum of NT‐proBNP was associated with thromboembolic events and all‐cause mortality in patients with ACS and/or AF.^8^, ^9^, ^10^ However, the predictive value of combined serum NT‐proBNP to CHA_2_DS_2_‐VASc score was unknown. Thus, in the present study, we sought to evaluate whether adding baseline NT‐proBNP level to CHA_2_DS_2_‐VASc score could improve risk stratification remarkably for patients with ACS and AF.

## 2 METHODS

### 2.1 Study population

The study population was derived from a prospective, real‐world, single‐center registry that enrolled consecutive, real‐world patients undergoing percutaneous coronary intervention (PCI) for coronary artery disease between January 2016 and December 2019 in the General Hospital of Northern Theater Command. The inclusion criteria were ACS patients presenting with AF and receiving PCI who were aged ≥18 years in the study. The major exclusion criteria were CHA_2_DS_2_‐VASc score cannot be calculated owing to incomplete data. Blood samples were obtained for measurement of NT‐proBNP levels with the automatic analyzer (Roche Cobas E411) with a normal range of NT‐proBNP < 125 pg/mL after admission for angiography. The choice of treatment was at the discretion of the physician. The protocol was approved by the ethics committee of the General Hospital of Northern Theater Command. The study complied with the provisions of the Declaration of Helsinki. Written informed consent was obtained from all participants.

### 2.2 Outcomes

The primary outcome was all‐cause death at 12 months. The secondary outcomes included 12‐month cardiac death and major adverse cardiovascular and cerebrovascular event (MACCE), defined as a composite of all‐cause death, myocardial infarction, or stroke. Clinical follow‐up was performed by phone call, out‐patient visit, or readmission by professional research staff at 3, 6, 9, and 12 months. A clinical events committee examined all clinical events.

### 2.3 Statistical analysis

The enrolled patients were divided based on the tertiles of NT‐proBNP. Continuous variables were presented as mean ± standard deviation or median (interquartile range [IQR]) and compared by analysis of variance or the Kruskal–Wallis test. Categorical variables were presented as frequencies and percentages and compared using *χ* ^2^ tests or Fisher exact tests. Time‐to‐event outcomes were analyzed by the Kaplan–Meier method, and compared by the log‐rank test. The Cox proportional hazard regressions were performed to calculate the hazard ratio (HR) and 95% confidence interval (CI) and evaluate the associations between NT‐proBNP and clinical outcomes. Variables selected for adjusted in the multivariable model were patient characteristics including age, gender, body mass index, hypertension, diabetes, a history of MI, prior PCI, prior stroke, and tobacco use; clinical factors at presentation including the type of ACS, hemoglobin, platelet, low‐density lipoprotein, renal function, and left ventricular ejection fraction; and procedure characteristics including access site, location of target vessels, number of stents, the total length of the stent, and stent diameter. To assess the added prognostic value of NT‐proBNP to CHA_2_DS_2_‐VASc score for all‐cause death and MACCE, receiver operator characteristic curves (ROCs) with the area under the curve (AUC), continuous net reclassification improvement (NRI), and integrated discrimination improvement (IDI) were performed. Comparisons of ROC were performed as described by DeLong et al. Unless otherwise specified, a two‐sided *p* < .05 was considered to indicate statistical significance. Statistical analysis was performed using SAS software version 9.4 (SAS Institute).

## 3 RESULTS

### 3.1 Study population

A total of 1223 patients with available baseline NT‐proBNP data were included in the final analysis. The median NT‐proBNP level was 1013.0 pg/mL (IQR, 342.8–2373.0 pg/mL). The baseline characteristics of the study population across quartiles of NT‐proBNP are shown in Table 1. Patients with higher NT‐proBNP levels were older and had a history of stroke and PCI. Those patients were also more likely to ST‐segment elevation myocardial infarction and had lower ejection fraction, estimated glomerular filtration rate, and hemoglobin. The CHA_2_DS_2_‐VASc score ranged from 0 to 9, with a median of 3 (IQR, 2–4). (Table 1) The number of stents implantation in the study cohort was 1.8 ± 1.1 with a total length of 47.4 mm per patient. The group with higher NT‐proBNP levels had a lower rate of radial artery access. There were also a few differences in medications during hospital and at discharge across the groups (Table 2).

**Table 1:** Baseline characteristics according to the tertiles of NT‐proBNP.

	Lower tertile (*N* = 407)	Middle tertile (*N* = 407)	Upper tertile (*N* = 409)	*p* value
Age, years	64.5 ± 9.2	67.5 ± 9.4	70.1 ± 10.1	<.001
Male	314 (77.2%)	301 (74.0%)	305 (74.6%)	.53
BMI, kg/m^2^	25.9 ± 3.4	26.3 ± 14.6	25.3 ± 10.0	.38
Hypertension	282 (69.3%)	279 (68.5%)	271 (66.3%)	.63
Diabetes	126 (31.0%)	133 (32.7%)	128 (31.3%)	.86
Previous MI	81 (19.9%)	99 (24.3%)	110 (26.9%)	.06
Previous stroke	53 (13.0%)	83 (20.4%)	90 (22.0%)	.002
Previous PCI	147 (36.1%)	126 (31.0%)	110 (26.9%)	.017
Smoking				.58
Active	151 (37.2%)	147 (36.2%)	159 (39.2%)	
Former	95 (23.4%)	83 (20.4%)	79 (19.5%)	
Never	160 (39.4%)	176 (43.3%)	168 (41.4%)	
Presentation				<.001
Unstable angina	319 (78.4%)	235 (57.7%)	127 (31.1%)	
NSTEMI	44 (10.8%)	73 (17.9%)	106 (25.9%)	
STEMI	44 (10.8%)	99 (24.3%)	176 (43.0%)	
LVEF, %	59.9 ± 7.4	54.5 ± 8.8	47.9 ± 10.0	<.001
eGFR, mL/min/1.73 m^2^ a	98.3 ± 25.1	89.3 ± 26.0	75.9 ± 27.5	<.001
Platelet count, 10^*9*^/L	212.9 ± 47.9	204. 9 ± 53.8	198.7 ± 61.8	<.001
Hemoglobin, g/dL	138.1 ± 16.3	136.0 ± 17.1	132.0 ± 18.5	<.001
LDL‐C, mmol/L	2.1 ± 0.7	2.3 ± 0.8	2.3 ± 0.7	.019
HDL‐C, mmol/L	1.1 ± 0.2	1.1 ± 0.2	1.1 ± 0.3	0.85
NT‐proBNP, pg/mL	180.5 (86.5–342.8)	1009.0 (765.9–1278.0)	3359.0 (2368.0–5315.0)	<.001
CHA_2_DS_2_‐VASc score	2.8 ± 1.6	3.3 ± 1.8	3.7 ± 1.6	<.001

*Note*: Data are mean ± standard deviation or median (interquartile range) or *n* (%).

Abbreviations: BMI, body mass index; eGFR, estimated glomerular filtration rate; HDL‐C, high‐density lipoprotein cholesterol; LDL‐C, low‐density lipoprotein cholesterol; LVEF, left ventricular ejection fraction; MI, myocardial infarction; NSTEMI, non‐ST‐segment elevation myocardial infarction; NT‐proBNP, N‐terminal pro‐B type natriuretic peptide; PCI, percutaneous coronary intervention; STEMI, ST‐segment elevation myocardial infarction.

^a^ Calculated using the Modification of Diet in Renal Disease formula.

**Table 2:** Procedural and medication results according to the tertiles of NT‐proBNP.

	Lower tertile (*N* = 407)	Middle tertile (*N* = 407)	Upper tertile (*N* = 409)	*p* value
Transradial access	380 (93.4%)	372 (91.4%)	355 (86.8%)	.0045
Target coronary artery				
Left main	36 (9.0%)	39 (9.7%)	42 (10.5%)	.78
Left anterior descending	197 (49.4%)	211 (52.6%)	206 (51.5%)	.65
Left circumflex	109 (27.3%)	91 (22.7%)	93 (23.3%)	.25
Right	158 (39.6%)	159 (39.6%)	161 (40.3%)	.98
Stents per patient	1.8 ± 1.0	1.7 ± 1.0	1.8 ± 1.1	.94
Stent length per patient, mm	47.4 ± 30.9	46.3 ± 30.4	48.4 ± 33.7	.67
Stent diameter, mm	3.1 ± 0.4	3.0 ± 0.4	3.0 ± 0.4	.28
Medication in‐hospital				
Aspirin	401 (98.5%)	397 (97.5%)	393 (96.1%)	.09
P2Y12 inhibitor				
Clopidogrel	358 (88.0%)	365 (89.7%)	372 (91.0%)	.37
Ticagrelor	119 (29.2%)	91 (22.2%)	96 (23.5%)	.052
Statin	404 (99.3%)	405 (99.5%)	396 (96.8%)	.002
ACEI/ARB	289 (71.0%)	274 (67.3%)	268 (65.5%)	.23
Beta‐blocker	319 (78.4%)	336 (82.6%)	354 (86.6%)	.009
Medication at discharge				
Aspirin	389 (96.0%)	372 (92.8%)	370 (91.6%)	.029
P2Y12 inhibitor				
Clopidogrel	312 (77.0%)	335 (83.5%)	339 (83.9%)	.018
Ticagrelor	100 (24.7%)	71 (17.7%)	71 (17.6%)	.015
Statin	395 (97.3%)	395 (98.3%)	394 (97.5%)	.63
ACEI/ARB	287 (70.5%)	284 (69.8%)	287 (70. 2%)	.97
Beta‐blocker	299 (73.7%)	327 (81.3%)	326 (80.7%)	.013

*Note*: Data are *n* (%).

Abbreviations: ACEI, angiotensin‐converting enzyme inhibitors; ARB, angiotensin II receptor blockers; NT‐proBNP, N‐terminal pro‐B type natriuretic peptide.

### 3.2 Clinical outcomes

**Figure 1:**
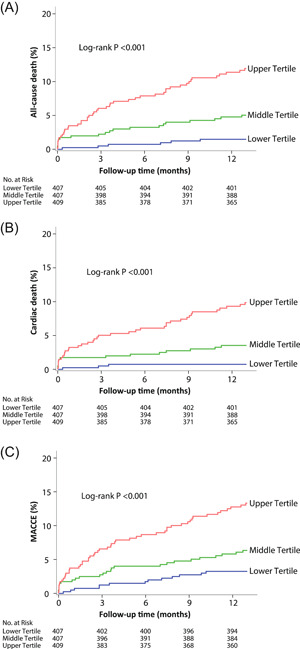
Kaplan–Meier curves for the primary and secondary endpoints across N‐terminal pro‐B‐type natriuretic peptide quartiles. (A) All‐cause death; (B) cardiac death; and (C) MACCE. MACCE denotes major adverse cardiac and cerebrovascular event, a composite of all‐cause death, myocardial infarction, or stroke.

Follow‐up at 12 months was completed in 1220 (99.7%) patients in the analysis. The principal outcomes are shown in Table 3. The primary outcome of 12‐month all‐cause death occurred in 6 (1.5%), 20 (4.9%), and 46 (11.2%) in the lower, middle, and upper tertile of NT‐proBNP levels group, respectively (log‐rank *p* < .001). There was a significant difference in the frequency of cardiac death (0.7% vs. 3.4% vs. 9.3%, log‐rank *p* < .001) and MACCE (3.2% vs. 6.1% vs. 12.5%, log‐rank *p* < .001) at 12 months across tertiles of NT‐proBNP. The Kaplan–Meier curves are shown in Figure 1. Multivariable analyses showed that higher NT‐proBNP levels were strongly associated with increased risks of all‐cause death (adjusted HR: 1.05, 95% CI, 1.03–1.07), cardiac death (adjusted HR: 1.05, 95% CI, 1.03–1.07), and MACCE (adjusted HR: 1.04, 95% CI, 1.02–1.06).

**Table 3:** Cox regression analysis of the association between NT‐proBNP and endpoints.

NT‐proBNP level categorical/countinous	Number of patients with the event (%)	Crude HR (95% CI)	Crude *p* value	Adjusted HR (95% CI)	Adjusted *p* value
All‐cause death					
Lower tertile	6 (1.5%)	Reference	–		
Middle tertile	20 (4.9%)	3.40 (1.36–8.46)	.009	1.90 (0.69–5.22)	.21
Upper tertile	46 (11.2%)	8.04 (3.44–18.83)	<.001	4.86 (1.85–12.76)	.001
NT‐proBNP (per 500 pg/mL)	–	1.05 (1.04–1.07)	<.001	1.05 (1.03–1.07)	<.001
Cardiac death					
Lower tertile	3 (0.7%)	Reference	–		
Middle tertile	14 (3.4%)	4.74 (1.36–16.50)	.01	2.40 (0.57–10.06)	.23
Upper tertile	38 (9.3%)	13.27 (4.10–42.98)	<.001	8.37 (2.32–30.19)	.001
NT‐proBNP (per 500 pg/mL)	–	1.06 (1.04–1.07)	<.001	1.05 (1.03–1.07)	<.001
MACCE					
Lower tertile	13 (3.2%)	Reference	–		
Middle tertile	25 (6.1%)	1.96 (1.001–3.82)	.049	1.20 (0.56–2.56)	.64
Upper tertile	51 (12.5%)	4.11 (2.24–7.56)	<.001	2.21 (1.06–4.61)	.04
NT‐proBNP (per 500 pg/mL)	–	1.05 (1.03–1.06)	<.001	1.04 (1.02–1.06)	<.001

*Note*: Variables selected for adjusted in the multivariable model were patient characteristics including age, gender, body mass index, hypertension, diabetes, a history of myocardial infarction, prior percutaneous coronary intervention, prior stroke, and tobacco use; clinical factors at presentation including the type of acute coronary syndrome, hemoglobin, platelet, low‐density lipoprotein, renal function, and left ventricular ejection fraction; and procedure characteristics including access site, location of target vessels, number of stents, total length of stent, and stent diameter.

Abbreviations: CI, confidence interval; HR, hazard ratio; MACCE, major adverse cardiac and cerebrovascular events; NT‐proBNP, N‐terminal pro‐B‐type natriuretic peptide.

### 3.3 Predictive performance of NT‐proBNP

**Figure 2:**
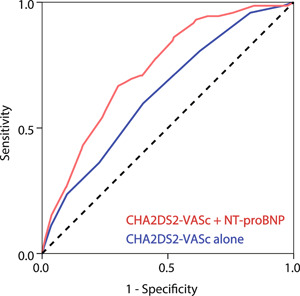
Receiver operating characteristic curves of the CHA_2_DS_2_‐VASc score and addition of N‐terminal pro‐B‐type natriuretic peptide (NT‐proBNP) to the CHA_2_DS_2_‐VASc score for all‐cause death.

The CHA_2_DS_2_‐VASc score showed moderate predictive value for 12‐month all‐cause death with AUC of 0.64 (95% CI: 0.68–0.70), cardiac death with AUC of 0.65 (95% CI: 0.58–0.72), and MACCE with AUC of 0.62 (95% CI: 0.56–0.68). When NT‐proBNP (quartiles) was combined with the score, there were significant improvements in discrimination and reclassification for prediction of all‐cause death (AUC: 0.73, 95% CI, 0.68–0.79, *p* = .001; NRI: 0.65, 95% CI, 0.42–0.88, *p* < .001; IDI: 0.03, 95% CI, 0.02–0.04, *p* < .001), cardiac death (AUC: 0.76, 95% CI, 0.70–0.82, *p* < .001; NRI: 0.75, 95% CI, 0.50–0.997, *p* < .001; IDI: 0.03, 95% CI, 0.02–0.04, *p* < .001), and MACCE (AUC: 0.69, 95% CI, 0.63–0.74, *p* = .007; NRI: 0.51, 95% CI, 0.30–0.73, *p* < .001; IDI: 0.02, 95% CI, 0.01–0.03, *p* < .001) (Table 4, Figure 2, and Supporting Information: Figure S1). The NT‐proBNP alone showed moderate predictive value for 12‐month all‐cause death with AUC of 0.69 (95% CI: 0.55–0.83), cardiac death with AUC of 0.72 (95% CI: 0.66–0.78), and MACCE with AUC of 0.65 (95% CI: 0.60–0.71). Compared with NT‐proBNP alone, NT‐proBNP combined with the CHA_2_DS_2_‐VASc score still had significant improvements for prediction of all‐cause death (*p* = .005), cardiac death (*p* = .009), and MACCE (*p* = .02).

**Table 4:** Additional prognostic value provided by NT‐proBNP beyond the CHA_2_DS_2_‐VASc risk score.

	AUC (95% CI)	*p* value	NRI (95% CI)	*p* value	IDI (95% CI)	*p* value
All‐cause death						
CHA_2_DS_2_‐VASc	0.64 (0.58–0.70)	–	Reference	–	Reference	–
CHA_2_DS_2_‐VASc + NT‐proBNP tertiles	0.73 (0.68–0.79)	.001	0.65 (0.42–0.88)	<.001	0.03 (0.02–0.04)	<.001
Cardiac death						
CHA_2_DS_2_‐VASc	0.65 (0.58–0.72)	–	Reference	–	Reference	–
CHA_2_DS_2_‐VASc + NT‐proBNP tertiles	0.76 (0.70–0.82)	<.001	0.75 (0.50–0.997)	<.001	0.03 (0.02–0.04)	<.001
MACCE						
CHA_2_DS_2_‐VASc	0.62 (0.56–0.68)	–	Reference	–	Reference	–
CHA_2_DS_2_‐VASc + NT‐proBNP tertiles	0.69 (0.63–0.74)	.007	0.51 (0.30–0.73)	<.001	0.02 (0.01–0.03)	<.001

Abbreviations: AUC, area under curve, CI, confidence interval; IDI, integrated discrimination improvement; MACCE, major adverse cardiac and cerebrovascular events; NRI, net reclassification improvement; NT‐proBNP, N‐terminal pro‐B‐type natriuretic peptide.

## 4 DISCUSSION

This study, using data from a large real‐world registry, was designed to evaluate the serum NT‐proBNP in combination with CHA_2_DS_2_‐VASc score to improve risk stratification for patients with ACS and AF. The principal findings from our study are that: (1) a higher plasma NT‐proBNP level at admission was associated with all‐cause death, cardiac death, and MACCE after adjustment for potential confounders; (2) the addition of NT‐proBNP improves risk discrimination for all‐cause death, cardiac death, and MACCE beyond the CHA_2_DS_2_‐VASc score during 12‐month follow‐up.

Individualized management strategy, as a medical model tailored to medical decisions based on the predicted risk of patients, is a key healthcare trend in contemporary clinical practice. In this regard, predicting algorithms had been developed for risk assessment and subsequent treatment advice. Previous studies have demonstrated that patients with concurrent ACS and AF appeared to be strongly associated with short‐ and long‐term mortality and thrombotic events.^3^, ^11^ Thus, it is essential to identify individuals at high risk and adopt proper therapeutic interventions to improve their prognosis. Generally, the CHA_2_DS_2_‐VASc score is a simple and well‐established scoring system to assess the risk of stroke and thromboembolism in patients with AF^6^ and extended to predict risk for other adverse events, such as HF and AF recurrence.^12^, ^13^ However, the risk of the coronary ischemic event also need to be carefully evaluated in patients with AF and ACS.^4^

The algorithm by adding biomarkers to the CHA_2_DS_2_‐VASc score might be a useful tool for physicians to identify the risk of coronary ischemic events in parallel to the routine clinical risk assessment of thromboembolism. The moderate discrimination ability for mortality and cardiac adverse events prediction is shown in our analysis, which is in‐agreement with a series of studies.^14^, ^15^, ^16^ This finding highlights the need for better risk stratification models in current clinical practice. Recently, a survey by the European Heart Rhythm Association revealed the variation and uncertainty in the interpretation of components of the CHA_2_DS_2_‐VASc score, especially the “C” component.^7^ Honestly, guideline education and updated and unified criteria might be effective strategies to improve the accuracy in scoring the CHA_2_DS_2_‐VASc score. However, it is notable that HF is by definition a clinical syndrome characterized by a series of symptoms and signs. The objective evidence for HF is also critical in diagnostic precision and treatment strategy, such as results from laboratory tests.^17^

NT‐proBNP is a versatile biomarker, as a non‐active prohormone released from the same molecule that produces BNP. The patients with higher values of NT‐proBNP are more susceptible to adverse events when compared with those with lower values.^9^, ^18^, ^19^ In addition, some evidence suggests serum NT‐proBNP could improve the predictability of risk models.^20^, ^21^, ^22^, ^23^ In the present study, we proved for the first time that NT‐proBNP can provide additional prognostic information beyond the CHA_2_DS_2_‐VASc score for all‐cause death, cardiac death, and MACCE, indicating NT‐proBNP as a value‐added biomarker to risk stratification in patients with concomitant ACS and AF. Admittedly, the inclusion of NT‐proBNP into the scoring algorithm has a potential risk to overestimate the influence of “C” element, congestive HF. However, the prognostic value of NT‐proBNP is independent of heart function in AF patients.^9^, ^24^, ^25^ The latent mechanism might be left atrium secretion.^26^ Furthermore, the concentration of NT‐proBNP could support physicians to identify patients with a high risk in the absence of clinical symptoms and signs. Indeed, more studies are warranted to confirm the position of NT‐proBNP in determining mortality and ischemic risk in AF patients with or without HF.

Limitations of our study should be considered. First, this is a post hoc analysis from a large prospective single‐center cohort of ACS patients undergoing PCI, which may affect the generalizability of our results. Further, specific‐designed studies are needed to confirm these findings. Second, the serum NT‐proBNP was measured at admission before the procedure. The temporal changes of NT‐proBNP might be better to predict long‐term risk. However, the multiple tests of biomarkers in risk assessment need to be balanced against cost‐effectiveness and complexity in routine clinical practice. Moreover, in the present study, adding baseline NT‐proBNP level to the CHA_2_DS_2_‐VASc score showed a robust prognostic value to assess long‐term adverse events. Finally, as with all risk‐scoring algorithms, a prospective evaluation would be desirable to investigate the clinical usefulness.

## 5 CONCLUSION

NT‐proBNP is a strong risk factor for poor prognosis and enables a more accurate appreciation of risk on top of the CHA_2_DS_2_‐VASc score in patients with co‐morbid ACS and AF. Whether the application of a scoring system could improve the identification of high‐risk patients and facilitate individualized therapeutic interventions needs further prospective evaluation.

## AUTHOR CONTRIBUTIONS

Xuefei Mu and Miaohan Qiu were responsible for writing the original draft, methodology, data curation, formal analysis, and visualization. Yi Li was responsible for conceptualization, methodology, data curation, project administration, and review editing. Ziqi Li, Bin Qi, and Zilan Jing were responsible for the investigation and review editing. Quanmin Jing was responsible for conceptualization, funding acquisition, data curation, project administration, methodology, supervision, and review editing.

## CONFLICT OF INTEREST STATEMENT

The authors declare no conflict of interest.

## Supporting information

Supporting information.

## Data Availability

The datasets used and analyzed in this study are available from the corresponding authors upon reasonable request.
