# Supplementary material for: N‐terminal pro‐B‐type natriuretic peptide improves the predictive value of CHA2DS2‐VASc risk score for long‐term cardiovascular events in acute coronary syndrome patients with atrial fibrillation
Source: Clin Cardiol. 2023 May 22;46(7):810–7. doi: 10.1002/clc.24037 (PMC10352965; doi:10.1002/clc.24037)
Supplement: Supplementary file 1 — Supporting information. [file CLC-46-810-s001.docx]

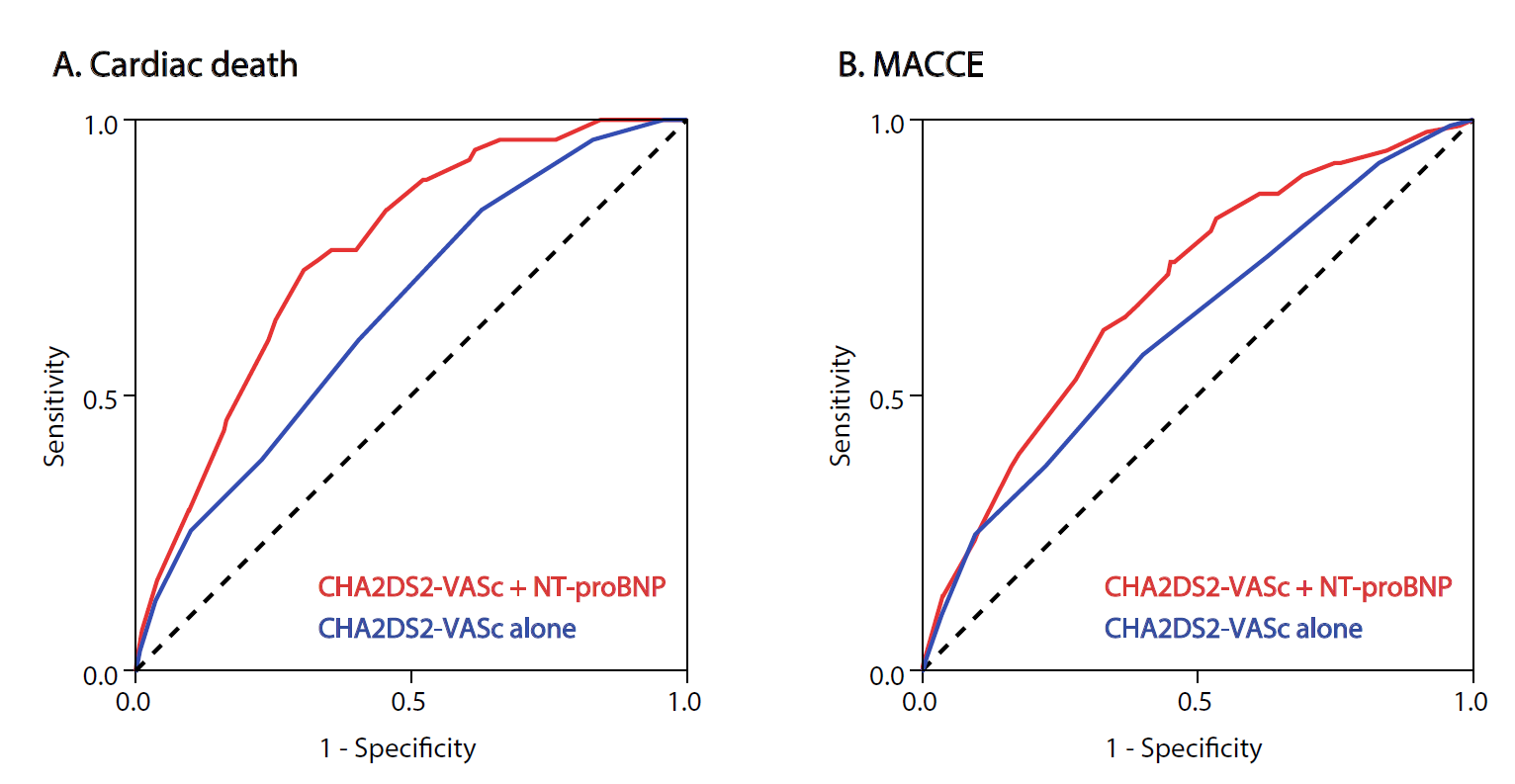


**Figure S1. Receiver operating characteristic curves of the CHA2DS2-VASc score and addition of NT-proBNP to the CHA2DS2-VASc score for A) Cardiac death; B) MACCE.**

MACCE denotes major adverse cardiac and cerebrovascular event, a composite of all-cause death, myocardial infarction, or stroke
